# Supplementary material for: Mapping the evolution of fertility support policies in China: A content and instrumental analysis
Source: PLoS One. 2025 Oct 9;20(10):e0332137. doi: 10.1371/journal.pone.0332137 (PMC12510515; doi:10.1371/journal.pone.0332137)
Supplement: S1 Appendix — (ZIP) [file pone.0332137.s001.zip › S1 Appendix. 226 original policy documents/176-中长期青年发展规划(2016-2025年)(FBM-CLI-16-292980).doc]

中长期青年发展规划(2016－2025年)

**发布部门：** [中国共产党中央委员会](https://www.pkulaw.com/law/chl?Aggs.IssueDepartment=a01) [国务院](https://www.pkulaw.com/law/chl?Aggs.IssueDepartment=5)

**发布日期：**2017.04

**实施日期：**2017.04

**时效性：** [现行有效](https://www.pkulaw.com/law/chl?Aggs.TimelinessDic=01)

**效力级别：** [党内法规制度](https://www.pkulaw.com/law/chl?Aggs.EffectivenessDic=XR12)

**法规类别：** [老少妇幼残保护](https://www.pkulaw.com/law/chl?Aggs.Category=01604)

中长期青年发展规划（2016－2025年）
（中共中央、国务院　2017年4月）

　　青年是国家的未来、民族的希望。青年兴则民族兴，青年强则国家强。促进青年更好成长、更快发展，是国家的基础性、战略性工程。依据党和国家有关政策法规，按照经济社会发展的总体目标和要求，结合我国青年发展的实际情况，制定本规划。
　　本规划所指的青年，年龄范围是14－35周岁（规划中涉及婚姻、就业、未成年人保护等领域时，年龄界限依据有关法律法规的规定）。

　　序言

　　党和国家历来高度重视青年、关怀青年、信任青年，始终坚持把青年作为党和人民事业发展的生力军，为青年在革命、建设、改革中施展才华创造条件、提供舞台；尊重青年敢想敢干、富有梦想的特质，注重激发青年的参与热情和创新活力，引领青年勇开风气之先、走在时代前列；关心、解决青年的现实问题和迫切需求，支持青年在人民的伟大奋斗中实现自己的人生理想。党的十八大以来，以习近平同志为核心的党中央高度重视青年发展事业，反复强调青年一代有理想、有担当，国家就有前途，民族就有希望，实现中华民族伟大复兴就有源源不断的强大力量；进一步明确中国特色社会主义青年运动方向，全面加强对青年的思想政治引领和成长成才服务，制定实施一系列促进青年发展的政策措施，激励引导青年与民族同命运、与祖国共奋进、与时代齐发展，为广大青年指明了正确成长道路，创造了良好成长环境。
　　在党和国家的关心、支持和推动下，我国青年发展事业取得巨大进步和历史性成就。青年的思想政治面貌总体健康向上，拥护中国共产党的领导，对中国特色社会主义事业充满信心；青年的基本生活条件不断改善，物质生活水平显著提高，精神文化生活日益丰富，青年群体文明程度不断提升；教育事业长足发展，青壮年人口文盲基本消除，新增劳动力平均受教育年限达到13.3年，处于我国历史上最好水平，与发达国家之间的差距显著缩小；社会保障制度更加健全、水平不断提升，法治国家建设不断推进，青年发展权益得到更好维护；青年的创新能力、创业活力不断增强，青年人才队伍不断壮大，在报效祖国、服务人民、奉献社会的过程中实现着自身的成长发展。
　　未来10年，是实现“两个一百年”奋斗目标、实现中华民族伟大复兴中国梦的关键时期。面对复杂多变的国际环境和国内艰巨繁重的改革发展任务，统筹推进“五位一体”总体布局和协调推进“四个全面”战略布局，适应和引领经济发展新常态，牢固树立和贯彻落实创新、协调、绿色、开放、共享的发展理念，需要青年一代充分发挥作用，在改革发展稳定第一线建功立业、接续奋斗。
　　青年是国家经济社会发展的生力军和中坚力量。党和国家事业要发展，青年首先要发展。必须清醒认识到，青年发展事业与社会主义现代化建设的新要求、经济社会发展的新形势、广大青年的新期待相比，还存在不少亟待解决的突出问题。主要是：青年思想教育的时代性、实效性有待增强，用共产主义和中国特色社会主义引领青年，用中国梦和社会主义核心价值观凝聚共识、汇聚力量的任务尤为紧迫；青年体质健康水平亟待提高，部分青年心理健康问题日益凸显；青年社会教育和实践教育需要加强，提高教育质量的任务仍十分艰巨；青年就业的结构性矛盾比较突出，影响就业公平的障碍有待进一步破除；青年创业创新的热情有待进一步激发，鼓励青年创业创新的政策和社会环境需要不断优化；人口结构的新特点新变化使得青年一代的工作和生活压力不断增大，在婚恋、社会保障等方面需要获得更多关心和帮助；统筹协调青年发展工作的体制机制还不完善，各方面共同推进青年发展的合力有待进一步形成。
　　赢得青年才能赢得未来，塑造青年才能塑造未来。要站在党和国家事业后继有人、兴旺发达的高度，把青年发展摆在党和国家工作全局中更加重要的战略位置，整体思考、科学规划、全面推进，努力形成青年人人都能成才、人人皆可出彩的生动局面，为实现“两个一百年”奋斗目标、实现中华民族伟大复兴的中国梦注入强劲、持久的青春动力。
　　**一、**指导思想、根本遵循、总体目标
　　1.指导思想。高举中国特色社会主义伟大旗帜，全面贯彻党的十八大和十八届三中、四中、五中、六中全会精神，坚持以马克思列宁主义、毛泽东思想、邓小平理论、“三个代表”重要思想、科学发展观为指导，深入学习贯彻习近平总书记系列重要讲话精神和治国理政新理念新思想新战略，坚持党管青年原则，牢牢把握为实现中华民族伟大复兴中国梦而奋斗的时代主题，充分照顾青年的特点和利益，优化青年成长环境，服务青年紧迫需求，维护青年发展权益，促进青年全面发展，引导青年树立共产主义远大理想和中国特色社会主义共同理想，坚定中国特色社会主义道路自信、理论自信、制度自信、文化自信，自觉团结凝聚在党的周围，更好成长为中国特色社会主义事业的合格建设者和可靠接班人。
　　2.根本遵循。坚持马克思主义青年观和中国特色社会主义青年运动方向，全面贯彻落实以习近平同志为核心的党中央关于青年工作的决策部署，引导广大青年坚定不移听党话、跟党走；坚持以青年为本，尊重青年主体地位，把服务与成才紧密结合，让青年有更多获得感，促进青年在投身实现中华民族伟大复兴中国梦的实践中放飞青春梦想、实现全面发展；坚持全局视野，从战略高度看待青年发展事业，党委加强领导，政府、群团组织、社会等各方面协同施策，共同营造有利于青年发展的良好环境。
　　3.总体目标。到2020年，具有中国特色的青年发展政策体系和工作机制初步形成，广大青年思想政治素养和全面发展水平进一步提升，在决胜全面建成小康社会伟大实践中的生力军和突击队作用得到充分发挥。到2025年，具有中国特色的青年发展政策体系和工作机制更加完善，广大青年思想政治素养和全面发展水平明显提升，不断成长为志存高远、德才并重、情理兼修、勇于开拓，堪当实现中华民族伟大复兴中国梦历史重任的有生力量。

　　**二、**发展领域、发展目标、发展措施
　　（一）青年思想道德
　　发展目标：广大青年积极践行社会主义核心价值观，中国特色社会主义道路自信、理论自信、制度自信、文化自信进一步增强，思想道德水平和文明素质进一步提高，为实现中国梦而奋斗的共同思想道德基础更加巩固。
　　发展措施：
　　1.加强青年理想信念教育。深入开展共产主义、中国特色社会主义和中国梦学习宣传教育，开展习近平总书记系列重要讲话精神和治国理政新理念新思想新战略学习教育，使中国梦成为青年共同追求的奋斗目标，使中国特色社会主义成为青年衷心拥护的发展道路，使共产主义成为青年矢志追求的远大理想，增进青年对党的信赖、信念、信心。注重引导青年学习马克思主义基本原理，树立辩证唯物主义和历史唯物主义的世界观、方法论。注重加强宣传教育、示范引领和实践养成，引导广大青年增强使命意识和责任意识，自觉把人生追求融入党和国家事业。深入实施青年马克思主义者培养工程。充分发挥思想政治理论课在青年学生思想政治教育中的主渠道作用。实施高校思想政治理论课建设体系创新计划，建设学生真心喜爱、终身受益的高校思想政治理论课。
　　2.在青年中培育和践行社会主义核心价值观。引导青年勤学、修德、明辨、笃实，使社会主义核心价值观内化为青年的坚定信念，外化为青年的自觉行动。大力弘扬以爱国主义为核心的民族精神和以改革创新为核心的时代精神，把爱国主义教育贯穿国民教育和精神文明建设全过程，引导青年学习了解党史国史、近现代史和改革开放史，继承五四运动以来的革命文化传统，坚持爱国、爱党、爱社会主义相统一，自觉培养爱国之情、砥砺强国之志、实践报国之行。引导青年传承弘扬中华优秀传统文化，增强文化自信和价值观自信。深入开展形式多样的青年群众性精神文明创建活动，引导青年大力弘扬社会公德、职业道德、家庭美德，培养良好个人品德，积极倡导和培育诚信品格，争当“向上向善好青年”，在引领社会文明风尚中发挥积极作用。加强民族团结宣传教育，推动各族青年交往交流交融，树立正确的国家观、民族观、历史观、文化观、宗教观，自觉抵制宗教极端思想，共同维护祖国统一和各民族繁荣发展。开展青年国防教育，推动军地青年共建共育，教育适龄青年自觉履行兵役义务。
　　3.分类开展青年思想教育和引导。面向中学中职学生，广泛开展“与人生对话”主题活动，引导他们从小确立人生奋斗的远大志向，培养爱国、爱党、爱社会主义的感情。面向大学生，广泛开展“与信仰对话”主题活动，引导他们认识马克思主义的真理性，坚定走中国特色社会主义道路的信念。面向企业青年，广泛开展岗位建功活动，引导他们正确看待个人、企业、社会、国家的关系，以积极、务实、理性的态度面对职业生涯中遇到的具体问题。面向进城务工青年，注重把解决思想问题与解决实际问题相结合，在排忧解难、传递关怀中引导他们心向党和政府、矢志拼搏奋斗。面向农村青年，广泛宣传党和政府的支农惠农政策，引导他们树立“农村天地广阔、青年大有可为”的思想认识。
　　4.强化网上思想引领。把互联网作为开展青年思想教育的重要阵地，团结、带动和壮大网上积极力量，大力开展正面宣传，实施“青年好声音”系列网络文化行动，增强网络正能量，消解网络负能量。提升网络舆情分析和引导能力，疏导青年情绪，澄清误解和谣言，引导青年形成正确认知。在青年群体中广泛开展网络素养教育，引导青年科学、依法、文明、理性用网。广泛开展青年网络文明志愿者行动，组织动员广大青年注册成为网络文明志愿者，参与监督和遏止网上各种违法和不良信息传播，为构建清朗网络空间作贡献。
　　（二）青年教育
　　发展目标：青年受教育权利得到更好保障，基本公共教育服务均等化逐步实现，教育公平程度明显提升。新增劳动力平均受教育年限达到13.5年以上，高等教育毛入学率达到50%以上。
　　发展措施：
　　1.提高学校育人质量。坚持立德树人，深化教育改革，把增强学生社会责任感、法治意识、创新精神、实践能力作为重点任务贯彻到学校教育全过程。改善课堂教学，调动青年学生自主学习的积极性，完善知识结构，培养创新兴趣和科学素养。科学设计开展实践育人活动，通过探索实施高校共青团“第二课堂成绩单”制度等途径，帮助学生开阔视野、了解社会、提升综合素质。丰富学生创新实践平台，深入开展“挑战杯”竞赛和中国青少年科技创新奖评选，支持培育学生科技创新社团，营造校园科技创新氛围，为学生开展科技创新探索提供必要条件。将中小学共青团、少先队工作纳入教育督导。完善现代职业教育体系，推进产教融合、校企合作，办好全国职业院校技能大赛。深化考试招生制度改革，把促进学生健康成长成才作为改革的出发点和落脚点，扭转片面应试教育倾向。加强教师队伍建设，严格教师准入制度，突出教师职业道德教育和业务能力培训，深化教师评价管理体系改革。深入开展文明校园、绿色校园创建，创造和谐优美校园环境。在社会科学研究机构、高等学校加强青年学研究。
　　2.科学配置教育资源。加大公共教育投入向中西部和民族边远贫困地区的倾斜力度，逐步缩小地区间教育资源差距。普及高中阶段教育，逐步分类实现中等职业教育全部免除学杂费，率先从建档立卡的家庭经济困难学生实施普通高中免除学杂费。实施国家贫困地区定向招生专项计划。完善贫困家庭学生、进城务工青年、少数民族青年和残疾青年等特殊青年群体帮扶救助机制，健全资助体系、完善资助方式，实现家庭经济困难学生资助全覆盖。进一步完善和落实进城务工人员随迁子女接受义务教育后在当地参加升学考试政策。
　　3.强化社会实践教育。完善扶持政策，加大经费投入，加强青年社会实践基地建设，鼓励机关、军队、企事业单位、社会组织为有组织的青年社会实践提供帮助和便利。在青年中广泛开展科普教育和群众性科技创新活动，引导广大青年讲科学、爱科学、学科学、用科学。广泛开展大中专学生“三下乡”、志愿服务等社会实践活动，鼓励青年参与社会公共服务和社会公益事业。推进青年信用体系建设，逐步应用到青年入学、就业、创业等领域，引导青年践行诚信理念。
　　4.促进青年终身学习。强化家庭教育基础作用，全面宣传普及家庭教育科学理念、知识和方法，实现家庭教育对优秀传统文化、爱国主义、社会责任、生活技能、勤俭美德、自律能力的基础性培养。大力发展继续教育，建立个人学习账号和学分累计制度，开展师生互动式、同伴共享式技能学习培训。加大青年社会教育投入，建立多渠道筹措资金投入机制。创造社会教育良好环境，规划青年成长成才各个环节的教育需求，统筹协调文化、出版、影视、网络等资源，实现对青年教育空间的全覆盖。构建并推行终身职业技能培训制度。推动各类学习资源开放共享，鼓励社会力量和民间资本提供多样化教育服务，推进教育信息化，发展在线教育和远程教育，扩大优质教育资源覆盖面，构建灵活开放的终身教育培训体系。
　　5.培育青年人才队伍。实施青年英才开发计划，在重点学科领域培养扶持一批青年拔尖人才；在高水平研究型大学和科研院所优势基础学科建设一批国家青年英才培养基地。统筹推进党政人才、企业经营管理人才、专业技术人才、高技能人才、农村实用人才、社会工作人才等领域青年人才队伍建设。建立健全对青年人才普惠性支持措施。加大教育、科技和其他各类人才工程项目对青年人才培养支持力度，在国家重大人才工程项目中设立青年专项。改革完善青年人才管理体制，创新青年人才培养开发、评价发现、选拔任用、流动配置、激励保障机制，善于发现、重点支持、放手使用青年优秀人才。加强知识产权保护，鼓励青年人才创新创造。鼓励和支持青年人才参与战略前沿领域研究，着力培养一批青年科技创新领军人才。坚持自主培养开发与海外引进并举，用好国内优秀人才，吸引海外高层次青年人才和急需紧缺青年专门人才。
　　（三）青年健康
　　发展目标：持续提升青年营养健康水平和体质健康水平，青年体质达标率不低于90%；有效控制青年心理健康问题发生率，青年心理健康辅导和服务水平得到较大提升；引领青年积极投身健康中国建设。
　　发展措施：
　　1.提高青年体质健康水平。实施全民健身计划，严格执行《[国家体育锻炼标准](https://www.pkulaw.com/chl/6caa4f0165ffff5dbdfb.html)》和《国家学生体质健康标准》，在学校教育中强化体质健康指标的硬约束。加强学校体育工作，完善国家体育与健康课程标准，发挥学校体育考核评价体系的导向作用，保证体育课时和课外锻炼时间得到落实。组织青年广泛参与全民健身运动，培养体育运动爱好，提升身体素质，掌握运动技能，养成终身锻炼的习惯。在城乡社区建设更多适应青年特点的体育设施和场所，配备充足的体育器材，方便青年就近就便开展健身运动。鼓励和支持青年体育类社会组织发展，带动更多青年培养体育兴趣和爱好。
　　2.加强青年心理健康教育和服务。注重加强对青年的人文关怀和心理疏导，引导青年自尊自信、理性平和、积极向上，培养良好心理素质和意志品质。促进青年身心和谐发展，指导青年正确处理个人与他人、个人与集体、个人与社会的关系。加强对不同青年群体社会心态和群体情绪的研究、管控和疏导，引导青年形成合理预期，主动防范和化解群体性社会风险。加强青年心理健康知识宣传普及，提高心理卫生知晓率。支持各级各类青年专业心理辅导机构和社会组织建设，大力培养青年心理辅导专业人才。重点抓好学校心理健康教育，在高校、中学和职业学校普遍设置心理健康辅导咨询室，有条件的学校配备专职心理健康教育师资队伍。构建和完善青年心理问题高危人群预警及干预机制。加强源头预防，注重对青年心理健康问题成因的研究分析，及时识别青年心理问题高危人群，采取有效措施解决或缓解青年在学业、职业、生活和情感等方面的压力。
　　3.提高各类青年群体健康水平。重视服务残疾青年的专业康复训练，落实器材、场所等配套保障。解决农村地区、贫困地区、西部地区青年学生的营养健康问题。引导高校学生“走下网络、走出宿舍、走向操场”，养成健康文明的生活习惯。做好青年职业病的预防和治疗工作，大幅度降低在职青年职业病发生率。关注进城务工青年健康状况，开展健康监测。动员社会力量，通过志愿服务、慈善捐助等形式为青年群体提供有针对性的健康服务。
　　4.加强青年健康促进工作。编撰和出版有关生命教育的读物，引导青年尊重生命、热爱生活。定期组织青年参与公共场所安全演练，开展灾害逃生、伤害自护、防恐自救、互助互救等体验教育，增强青年在应对突发性事件中的自我保护意识和防灾避险能力。在青年中倡导健康生活方式，加强健康教育，提升青年健康素养水平。广泛开展禁烟宣传，让青年成为支持禁烟、自觉禁烟的主体人群。完善艾滋病和性病的防治工作机制，针对重点青年群体加强宣传教育，推广有效的干预措施，切实降低艾滋病和性病发生率。做好禁毒宣传教育工作，提高青年群体尤其是青年学生群体对毒品及其危害性的认识。强化对娱乐场所的监管，严厉打击吸毒贩毒、卖淫嫖娼等违法犯罪行为。
　　（四）青年婚恋
　　发展目标：青年婚恋观念更加文明、健康、理性；青年婚姻家庭和生殖健康服务水平进一步提升；青年的相关法定权利得到更好保障。
　　发展措施：
　　1.加强青年婚恋观、家庭观教育和引导。将婚恋教育纳入高校教育体系，强化青年对情感生活的尊重意识、诚信意识和责任意识，引导青年树立文明、健康、理性的婚恋观。发挥大众传媒的社会影响力，广泛传播正面的婚恋观念，鲜明抵制负面的婚恋观念，形成积极健康的舆论导向。倡导结婚登记颁证、集体婚礼等文明节俭的婚庆礼仪。引导青年树立正确的家庭观念，倡导尊老爱幼、男女平等、夫妻和睦、勤俭持家、邻里团结，传承优良家教家风，培育家庭文明。加强青年敬老、养老、助老道德建设，大力弘扬孝敬老人的传统美德。
　　2.切实服务青年婚恋交友。支持开展健康的青年交友交流活动，重点做好大龄未婚青年等群体的婚姻服务工作。规范已有的社会化青年交友信息平台，打造一批诚信度较高的青年交友信息平台。依法整顿婚介服务市场，严厉打击婚托、婚骗等违法婚介行为。充分发挥工会、共青团、妇联等群团组织和社会组织的作用，为青年婚恋交友提供必要的基础保障和适合青年特点的便利条件。
　　3.开展青年性健康教育和优生优育宣传教育。在青年中加强对国家人口发展战略和政策的宣传教育，促进人口均衡发展。加大对性知识的普及力度，在有条件的学校推广性健康课程，加强专兼职性健康教育师资队伍建设。预防和减少不当性行为对青年造成的伤害，大幅度降低意外妊娠的发生率。大力弘扬以“婚育文明、性别平等；计划生育、优生优育；生殖健康、家庭幸福”为核心的婚育文化，坚决抵制非医学需要的胎儿性别鉴定和选择性别人工终止妊娠行为。加大对适龄青年的婚育辅导力度，加大适龄青年婚前检查、孕前检查和产前检查的普及力度。
　　4.保障青年在孕期、产假、哺乳期期间享有的法定权益。全面落实女性青年在怀孕、生育和哺乳期间依法享有的各项权利。鼓励条件成熟的地方探索在物质、假期等方面给予青年更多支持。
　　（五）青年就业创业
　　发展目标：青年就业比较充分，高校毕业生就业保持在较高水平；青年就业权利保障更加完善，青年的薪资待遇、劳动保护、社会保险等合法权益得到充分保护；青年创业服务体系更加完善，创业活力明显提升。
　　发展措施：
　　1.推动完善促进青年就业创业政策体系。根据就业形势和就业工作重点变化，加强就业政策与产业、贸易、财税、金融等政策的协调，进一步完善积极就业政策。发挥公共财政促进青年就业作用，完善落实财政金融扶持政策，扶持发展现代服务业、战略性新兴产业、劳动密集型企业和小微企业，吸纳青年就业。加强对灵活就业、新就业形态的支持，促进青年自主就业，鼓励多渠道多形式就业。进一步完善青年创业就业配套政策及法律法规。加强就业统计工作，健全青年就业统计指标体系。
　　2.加强青年就业服务。实施青年就业见习计划。健全城乡均等的公共就业创业服务体系，完善服务功能，把有就业意愿的青年全部纳入服务范围，全面落实免费公共就业服务，对就业困难青年提供就业援助，帮助长期失业青年就业。创新就业信息服务方式方法，注重运用互联网技术打造适合青年特点的就业服务模式。加强青年职业培训，健全面向青年的劳动预备制培训计划，落实职业培训补贴政策。实施离校未就业高校毕业生就业促进计划，为毕业生提供职业指导、就业信息、就业见习、就业帮扶等服务。开展青年农民工职业技能培训，通过订单、定向和定岗式培训，对农村未升学初高中毕业生等新生代农民工开展就业技能培训，为有创业意愿的青年农民工提供创业培训。开展青年重点群体职业培训，加大贫困家庭子女、青年失业人员和转岗职工、退役青年军人和残疾青年等劳动者职业技能和创业培训力度，按规定提供培训补贴，对农村贫困家庭学员和城市居民最低生活保障家庭学员给予生活补贴。
　　3.推动青年投身创业实践。建立青年创业人才汇聚平台，建设青年创业导师团队，开展普及性培训和“一对一”辅导相结合的创业培训活动，帮助青年增强创业意识、增进创业本领。推动青年创业第三方综合服务体系建设，搭建各类青年创业孵化平台，完善政策咨询、融资服务、跟踪扶持、公益场地等孵化功能。加大青年创业金融服务落地力度，优化银行贷款等间接融资方式，支持创业担保贷款发展，拓宽股权投资等直接融资渠道。支持青年创业基金发展，发挥好国家新兴产业创业投资引导基金和中小企业发展基金等政府引导基金的作用，带动社会资本投入，解决青年创业融资难题。落实结构性减税和普遍性降费政策。建设青年创业项目展示和资源对接平台，搭建青年创业信息公共服务网络，办好青年创新创业大赛、展交会、博览会等创业品牌活动。着力培育服务青年创业的社会组织，建设专业化的服务队伍和服务实体。深入实施大学生创业引领计划，建立健全教学与实践相融合的高校创新创业教育体系，显著提升青年创新型人才培养质量；整合发展国家和省级高校毕业生就业创业基金。深入开展农村青年创业致富带头人培养，支持青年返乡创业。完善互联网创新创业政策，实施青年电商培育工程。加强对留学回国创业青年的服务，帮助他们了解国内信息、熟悉创业环境、交流创业经验、获得政策扶持。推动形成鼓励创新、宽容失败的体制机制和社会环境，更好激发青年创新潜能和创业活力。
　　4.加强青年就业权益保障。完善青年就业、劳动保障权益保护机制，加大劳动保障监察执法、劳动人事争议调解仲裁诉讼、安全生产监管监察工作力度。加强人力资源市场监管，规范招人用人制度，营造公平就业环境。完善失业保险、社会救助与就业的联动机制。
　　（六）青年文化
　　发展目标：更好引导青年传承中华优秀传统文化、弘扬社会主义先进文化。青年文化活动更加丰富，文化精品不断增多，传播能力大幅提升，人才队伍发展壮大，服务设施、机构和体制更加健全。青年对提升国家文化软实力贡献率显著提高。
　　发展措施：
　　1.加强文化精品创作生产。发挥精神文明建设“五个一工程”、国家舞台艺术精品创作工程、中国艺术节、中国文化艺术政府奖、中国新闻奖、中国出版政府奖等国家级重大工程项目、评奖的引导带动作用，鼓励文化机构、文艺工作者特别是青年文化人才，创作生产展现当代青年奋发向上、崇德向善、传承中华文明的文化精品。引领网络文化，保护网络文化知识产权，扶持高质量网络文化产品生产，加强微电影、动漫、游戏等内容创作创新，提升优秀网络文化产品供给能力和传播能力。国家艺术基金、国家出版基金等文化发展基金要加强对青年题材重点选题项目的扶持，鼓励优秀青年文化人才参与创作，支持青年题材优秀图书、影视、音乐、舞蹈、戏剧、曲艺、美术等生产、发行和推广。
　　2.丰富青年文化活动。广泛开展优秀文化作品全国性巡展巡演。深入挖掘中华优秀传统文化的时代价值，开展优秀传统文化艺术展示交流，引导青年积极参与文化遗产保护、传统工艺振兴、民间文艺传承。以校园文化、企业文化、军营文化、乡村文化、社区文化、社团文化、网络文化为载体，加强基层特色文化品牌建设，推动青年人均年度图书阅读量和艺术鉴赏、科普水平逐年提高。加强中国青年与各国青年人文交流，学习、吸收、借鉴世界优秀文化成果，讲好中国故事、传播好中国声音，不断提升文化自信。
　　3.造就青年文化人才。通过全国文化名家暨“四个一批”人才培养工程、文化产业人才培养工程、非物质文化遗产传承人、新闻出版广播影视领军人才和互联网创新人才培养等项目，实施青年文化人才培养计划，资助具备文化创新能力、掌握现代传媒技术、熟悉国际人文交流、善于经营管理的青年文化人才主持重大课题研究、领衔重点文化项目。加强后备文化人才队伍建设，面向青年文化工作者开展文化创意服务、文化生产实践、文化经营管理、媒体融合发展、国际合作规则等方面培训，凝聚文化研究、创作、表演、传播、经营、管理、志愿服务等青年人才。
　　4.优化青年文化环境。鼓励和支持有条件的报刊、电台、电视台、新闻网站设立青年栏目、节目，制作和传播有益于青年健康成长的内容，增加青年题材报道内容和播出时间，大力宣传青年在推动经济社会发展中的积极作用。在报刊和网络重点栏目、电视和院线黄金时段，增加优秀青年文化精品的宣传内容、频次，引导青年树立高尚精神追求、文明生活方式和正确消费观念。推进公共文化设施免费开放，增强针对青年群体的服务功能。
　　5.积极支持青年文化建设。加强文化理论研究，及时掌握青年文化需求、文化观念、文化潮流的动态变化，引领和指导青年文化实践。扶持以服务青年为主要功能的报纸、刊物、新闻出版、网站等文化企事业单位发展。完善公益性演出补贴制度，通过票价补贴、剧场运营补贴等方式，支持青年艺术表演团体公益演出。促进企业和民间资本增加对青年文化事业的投入。鼓励国家投资、资助或拥有版权的文化产品无偿用于公益性青年文化活动和服务。鼓励和支持各类文化单位在五四青年节面向青年免费或低收费开展文化活动、提供文化服务。采取政府购买、项目补贴、定向资助等方式，鼓励青年文化阵地、青年文化团体等社会力量承接青年文化服务。
　　（七）青年社会融入与社会参与
　　发展目标：青年更加主动、自信地适应社会、融入社会。青年社会参与的渠道和方式进一步丰富和畅通，实现积极有序、理性合法参与。共青团、青联、学联组织在促进青年社会融入和社会参与中的主导作用充分发挥，带动各类青年组织在促进青年有序社会参与中发挥积极作用。青年参与社会主义现代化建设的积极性主动性进一步增强，青年志愿服务水平进一步提高。不同青年群体相互理解尊重。青年对外交流合作不断拓展。
　　发展措施：
　　1.健全党领导下的以共青团为主导的青年组织体系。积极推进共青团改革，着力构建凝聚青年、服务大局、当好桥梁、从严治团的工作格局，充分发挥共青团作为党的助手的作用。加强共青团自身建设，适应青年发展的新情况新特点，不断创新组织设置，更多更广地覆盖新兴领域青年和流动青年；尊重青年主体地位，调动广大青年参与的积极性和主动性，活跃基层团组织，完善青年社会参与的基本组织依托。教育广大共青团员切实增强先进性光荣感，自觉做共产主义远大理想和中国特色社会主义共同理想的坚定信仰者和忠实实践者，充分发挥在青年中的模范作用和对青年的凝聚作用。充分发挥青联在爱国主义、社会主义旗帜下广泛团结各族各界青年的功能，强化共青团在青联组织中的引领作用，推动青联组织带领各族各界青年在大团结大联合中实现共同发展。加强共青团对学联组织的指导，推动学联组织引导学生追求进步、维护学生合法权益。发展培育青年社团，加强对各行各业青年的凝聚和服务。更好联系、服务和引导青年社会组织，促进青年有序社会参与。支持共青团、青联、学联依法承接政府职能转移，更好参与青年社会事务管理和服务；支持各类青年社会组织立足自身优势，以合适方式参与政府购买服务。
　　2.着力促进青年更好实现社会融入。鼓励和支持青年参与社会实践和公益服务，推动理论学习与劳动实践相结合，突出个人实践与社会公益有机统一，学会自我教育、自我管理、自我提升，在为家庭谋幸福、为他人送温暖、为社会作贡献的过程中增加人生历练，强化社会交往能力和社会责任感。充分发挥家庭在青少年社会融入中的重要作用，鼓励青少年自强自立，为青少年接触社会、开展社会交往创造更多机会、提供有效指导。学校教育要支持青年学生开展各种课外和校外活动，加强对青年学生社会融入的针对性指导，促进青年学生学会生存生活，学会做人做事，主动了解社会、适应社会。积极促进在内地就学、就业少数民族青年和进城务工青年及其子女的社会融入，帮助他们更快适应当地习俗、更好融入所在社区。充分发挥青年社会组织等社会力量的独特作用，吸引和带动青年广泛参与各类社会服务，不断培养和提升社会化技能。引导青年正确认识网络空间与现实社会的关系，多到社会实践中长见识、练本领，防止沉迷网络。要在全社会推动形成鼓励青年多样化参与、支持青年个性发展、宽容青年失误的氛围，为青年更好融入社会营造良好环境。
　　3.引领青年有序参与政治生活和社会公共事务。支持共青团、青联代表和带领青年积极参与人大、政府、政协、司法机关、社会有关方面各类协商，就涉及青年成长发展的重大问题协商探讨、提出意见、凝聚共识，充分发挥政治参与职能。探索建立有关人大代表、政协委员青少年事务联系机制，为青年参与畅通渠道、搭建平台。鼓励青年参与城乡基层群众自治，推动完善民主恳谈、民主议事制度，在实践中提高青年政治参与能力。推荐优秀青年代表担任人民陪审员、人民监督员、人民调解员等，依法履行相关职责。
　　4.鼓励青年在经济社会发展中充分发挥生力军和突击队作用。围绕国家整体发展战略需要，深化各类建功活动，树立先进典型，激励青年在各行各业积极创新，拓展工作领域和空间，形成发展新动力。鼓励青年积极参与生态环境保护，带头践行绿色生产生活方式，共建生态文明，共创美丽中国。组织动员广大青年积极投身脱贫攻坚，充分发挥青年企业家、青年科技工作者、青年致富带头人、青年志愿者等群体作用，为贫困地区改善区域发展环境、促进经济社会发展提供资金、人才、技术、管理等支持。摸清底数、精准施策，充分发挥教育和就业创业在青年脱贫中的重要作用，促进贫困青年早日脱贫。坚持围绕大局、服务社会需求、突出青年特色，深化青年志愿服务工作，组织引导广大青年大力弘扬“奉献、友爱、互助、进步”的志愿精神。
　　5.引导青年社会组织健康有序发展。加强对青年社会组织的政治引领，完善党委和政府与青年社会组织沟通交流机制，把对青年社会组织的管理和引导纳入法治化轨道。改进对青年社会组织的联系服务，充分发挥共青团和青联组织作用，通过资金支持、提供阵地场所、培训骨干人员等方式扶持青年社会组织健康发展。重点支持行为规范、运作有序、公信力强、适应经济社会发展要求的青年社会组织，重点发展科技类、公益慈善类、城乡社区服务类青年社会组织，积极发挥重点青年社会组织的示范带动作用。改善对青年社会组织的监督管理，建立完善民政部门和共青团、青联等群团组织及有关职能部门协同发挥作用的管理机制。
　　6.增进不同青年群体的交流融合。整合各方资源，帮助解决重点、新兴领域青年群体的实际困难，增进新生代农民工、青年企业家、青年社会组织骨干、青年新媒体从业人员、高校青年教师、归国留学青年等群体的政治认同和社会参与。发挥共青团组织优势，主动联系新的社会阶层青年群体，吸纳他们中的优秀分子进入组织体系。创造条件推动不同阶层、不同领域青年群体进行经常性对话交流，增进理解、认同和包容，舒缓社会压力，融洽社会关系。
　　7.增强港澳台青年的国家认同、民族认同和文化认同。实施港澳台青少年交流计划，以中华文化为纽带，不断探索创新工作方式，提高交流实效，实现在多元文化背景下包容差异、消除隔阂、增进认同。积极创造条件，搭建港澳台青年来内地创新创业平台，支持港澳台青年在国家发展及海峡两岸暨港澳经贸融合中寻找发展机会，为港澳台青年就业创业提供便利服务。帮助港澳台青年形成对“一国两制”的正确认知、对祖国文化的认同。
　　8.支持青年参与国际交往。拓宽青年参与国际交往的渠道，为青年开展国际交流与合作搭建更广阔的平台。完善选拔方式、丰富选拔手段，让更多的青年群体代表参与国际交流。培养推荐青年优秀人才到国际组织任职。加大宣传力度，提升青年国际交流活动的影响力和辐射面。
　　（八）维护青少年合法权益
　　发展目标：青少年权益维护的法律法规和政策体系更加完善，得到全面贯彻实施。青少年权益保护的工作体系和工作机制更加健全，合法权益得到切实维护。侵害青少年合法权益的行为受到有效打击和遏制。
　　发展措施：
　　1.全面贯彻实施有关青少年发展的法律法规。加强《[中华人民共和国未成年人保护法](https://www.pkulaw.com/chl/5b242b5a062cc53bbdfb.html)》、《[中华人民共和国预防未成年人犯罪法](https://www.pkulaw.com/chl/9a2ed33b50941af4bdfb.html)》以及教育、卫生、就业创业、社会保障等领域涉及青少年权益的法律法规贯彻实施，切实保障青年合法权益。共青团等群团组织要及时了解和研判青年发展状况，监督涉及青年发展权益的法律法规和政策执行，代表青年向有关部门反映问题、提出建议，推动及时有效解决青年实现发展权益面临的现实困难和突出问题。
　　2.完善青少年权益维护法律法规和政策。针对青年权益保障中的突出问题，制定修改相关法律法规和政策，在现有法律法规和政策体系中增加有利于维护青年普遍性权益的内容。以《[中华人民共和国未成年人保护法](https://www.pkulaw.com/chl/5b242b5a062cc53bbdfb.html)》、《[中华人民共和国预防未成年人犯罪法](https://www.pkulaw.com/chl/9a2ed33b50941af4bdfb.html)》和《[中华人民共和国刑事诉讼法](https://www.pkulaw.com/chl/6d5f42efc8724124bdfb.html)》中的“未成年人刑事案件诉讼程序”专章为基础，建立健全涵盖福利、保护、司法等内容的未成年人法律制度。加快制定电子商务、个人信息保护、互联网信息服务管理等法律法规，出台《未成年人网络保护条例》，严格落实互联网服务提供者的主体责任，有效防范暴力、色情、赌博、毒品、迷信、邪教等腐朽没落文化和丑化党和国家形象及革命先烈的信息传播。
　　3.健全青少年权益保护机制。尊重青年主体地位，拓展青年权益表达渠道，充分发挥共青团、青联组织代表和反映青年普遍性利益诉求的作用。建立青年权益状况舆情监测体系和舆论引导机制。支持共青团建设青少年维权工作网络平台和12355青少年服务台，把法治宣传教育与法律服务结合起来，带动青年社会组织、青少年事务社会工作者积极参与维护青少年权益。深化“青少年维权岗”创建活动，建立健全基层青少年维权工作机制。加强对困难青年群体、进城务工青年及其未成年子女等群体的关爱和权益维护工作。完善法律援助工作网络，鼓励和支持法律服务机构、社会组织、事业单位等依法为未成年人提供公益性法律服务和援助。健全未成年人行政保护与司法保护衔接机制，加强监护缺失、受到监护侵害的未成年人权益保护工作。
　　4.依法打击侵害青少年合法权益的行为。贯彻落实涉及青少年权益保护的法律法规，严厉打击拐卖、性侵害、遗弃、虐待等侵害未成年人合法权益的违法犯罪行为。大力开展青少年禁毒工作，依法惩处涉及青少年的毒品违法犯罪活动。严厉打击涉校违法犯罪活动。加强网络领域综合执法，严厉打击各类涉青少年网络违法犯罪。
　　（九）预防青少年违法犯罪
　　发展目标：青少年法治宣传教育常态化、全覆盖，青少年法治观念和法治意识不断增强，成长环境进一步净化。形成比较完善的重点青少年群体服务管理和预防犯罪工作格局，建立针对有严重不良行为和涉罪青少年进行教育矫治的有效机制，青少年涉案涉罪数据逐步下降。
　　发展措施：
　　1.加强法治宣传教育。在青少年中广泛开展法治宣传教育，使青少年明确基本的法律底线和行为边界，自觉尊法学法守法用法。把法治教育纳入国民教育体系，坚持课堂教学主渠道，积极开拓第二课堂，配齐配强中小学校兼职法治副校长、辅导员。落实国家机关“谁执法谁普法”普法责任制，建立法官、检察官、行政执法人员、律师在法律实施过程中面向青少年开展法治教育的制度规范。把法治教育纳入精神文明创建和平安建设内容，健全媒体公益普法制度，注重运用网络新媒体扩大宣传教育覆盖面，统筹青少年法治教育实践基地建设，发展壮大青少年普法工作队伍和志愿者队伍。
　　2.优化青少年成长环境。清理和整治社会文化环境，加大“扫黄打非”工作力度，打击各类侵权盗版行为，加强对影视节目的审查，强化以未成年人为题材和主要销售对象的出版物市场监管。加强校园周边环境治理和安全防范工作，严格落实禁止在中小学校园周边开办上网服务营业场所、娱乐场所、彩票专营场所等相关规定。依法采取必要惩戒措施，有效遏制校园欺凌、校园暴力等案（事）件发生。净化网络空间，完善网络文化、网络出版、网络视听节目审查制度和市场监管，定期开展专项整治行动，持续整治网络涉毒、淫秽色情及低俗信息。推动互联网上网服务行业健康发展，进一步规范上网服务营业场所服务管理，依法查处违规接纳未成年人的行为，依法取缔无照场所。
　　3.做好重点青少年群体服务管理工作。大力推进“为了明天”预防青少年违法犯罪工程，推动预防青少年违法犯罪工作列入各地工作规划和财政预算，不断健全组织机构和工作体系。在全国县级地区全面推开并不断深化重点青少年群体服务管理工作，明确各类群体工作重点，建立覆盖完整、切实有效、主责清晰、协调联动的工作机制。强化家庭监护和学校教育职责，防止青少年脱离与家庭、学校的联系，出现不良行为时能够及时采取有针对性的预防措施。加强专门学校建设和专门教育工作，畅通有严重不良行为未成年人进入专门学校接受教育矫治的渠道，研究建立符合条件的涉案未成年人进入专门学校接受教育矫治的程序。完善专门学校管理体制和运行机制，加强教师队伍建设，不断提高教育矫治水平。充分发挥青少年事务社会工作专业人才和社会工作服务机构作用，对重点青少年群体提供困难帮扶、法治教育、法律援助、心理疏导、行为矫治等专业服务。
　　4.完善未成年人司法保护制度。深化未成年人司法改革，公安机关、人民检察院、人民法院、司法行政机关要加强专门机制建设，明确专门机构或者指定专人办理未成年人违法犯罪案件。改革完善未成年人收容教养制度。在侦查、起诉、审判、刑事执行涉及未成年人案件中，落实社会调查、心理疏导与测评、分押分管、严格限制适用逮捕措施、强制辩护、合适成年人参与、当事人和解、附条件不起诉、分案起诉、法庭教育、回访帮教、犯罪记录封存、分类矫治等特殊保护制度。有条件的地区建立未成年人帮教基地。妥善安置附条件不起诉、适用非监禁刑、特赦的未成年人以及解除收容教养和其他刑满释放的青少年。
　　（十）青年社会保障
　　发展目标：社会保障体系充分覆盖青年急需的保障需求，并在各类青年群体之间逐步实现均等化。
　　发展措施：
　　1.加强对残疾青年的关心关爱和扶持保障。健全完善残疾青年教育、医疗、就业等方面的服务保障政策，进一步提高保障水平和服务能力。推动残疾青年平等参与社会生活、共享经济社会发展成果，依法保障残疾青年政治、经济、社会、文化教育权利。大力开展面向残疾青年的专业社会工作和志愿服务，鼓励和引导社会各界参与、支持残疾青年权益维护，培育理解、尊重、关心、帮助残疾青年的社会风尚。
　　2.加强青年社会救助工作。完善社会救助制度，健全救助服务管理工作机制。加大对流浪未成年人的救助力度，促使其回归家庭，有针对性地解决流浪未成年人在心理、健康、技能等方面存在的问题。为家庭困难的失学、失业、失管青年提供就业、就学、就医、生活等方面的帮助。加大临时救助政策的落实力度，解决包括进城务工青年在内的困难群众突发性、紧迫性、临时性生活困难。切实解决部分农村留守儿童中存在的学业失教、生活失助、亲情失落、心理失衡、安全失保问题。大力推进城镇基本公共服务向常住人口全覆盖，为进城务工青年与其未成年子女共同生活提供生活居住、日间照料、义务教育、医疗卫生等方面的帮助。

　　**三、**重点项目
　　1.青年马克思主义者培养工程。着重在青年学生骨干、团干部、青年知识分子等青年群体中选拔一批骨干作为培养对象，以理想信念教育为核心，开设党性教育、理论学习、实践锻炼、工作锤炼、对外交流等方面的课程，进行阶段性集中教育培训，着力培养一批对党忠诚、信仰坚定、素质优良、作风过硬的中国特色社会主义事业合格建设者和可靠接班人。注重后续跟踪培养，动态调整培养方式，充分发挥骨干力量对各行业的示范带动作用。青年马克思主义者培养工程分全国、省级、省级以下三级实施，每年培养不少于20万人。
　　2.青年社会主义核心价值观培养工程。坚持不懈用党的科学理论武装青年，推动邓小平理论、“三个代表”重要思想、科学发展观特别是习近平总书记系列重要讲话精神和治国理政新理念新思想新战略进课堂、进教材、进头脑，引导青年深入理解党的理论和路线方针政策。把社会主义核心价值观融入青年教育全过程，搭建课堂教学、社会实践、文化熏陶等多位一体的育人平台；开展革命传统教育和公民道德宣传，宣传先进青年典型，开展社会道德实践，引导青年形成修身律己、崇德向善、诚信互助、礼让宽容的道德风尚。引导青年传承弘扬中华优秀传统文化，深刻挖掘重要节庆日、纪念日蕴藏的丰富教育资源，引导青年汲取中华优秀传统文化的思想精华和道德精髓，增强做中国人的骨气和底气。
　　3.青年体质健康提升工程。深化学校体育改革，强化体育课和课外锻炼，以足球为突破口，集中打造青年群众性体育活动载体，大力开展阳光体育系列活动和大学生“走下网络、走出宿舍、走向操场”主题课外体育锻炼活动，使坚持体育锻炼成为青年的生活方式和时尚。培养青年体育运动爱好，经常性参加足球、篮球、排球、田径、游泳、乒乓球、羽毛球、网球等体育运动项目和健身操（舞）、健步走、传统武术、太极拳、骑车、登山、跳绳、踢毽等健身活动，力争使每个青年具备1项以上体育运动爱好，养成终身锻炼的习惯。引导青年树立健康促进理念，在健康促进事业中发挥积极作用。完善青年体质健康监测体系，实现定期抽样监测和公开发布监测结果，倡导青年形成良好的饮食、用眼和睡眠习惯，控制肥胖、近视、龋齿等常见病的发生率。改进普通高校高水平运动队招生工作，激励青年学生参与体育锻炼。
　　4.青年就业见习计划。按照“项目化运作、社会化动员、规范化管理”思路，在企业、社区、科研院所建设一批见习、实习基地，开发一批具有职业发展空间、技能训练机会的见习、实习岗位。把大学生实习纳入高校实践学分管理。把未就业大中专毕业生、各类社会青年纳入就业见习范围。加强青年就业见习培训和管理，提高见习实效。充分汇聚政府、企业、社会的力量，为青年参与就业见习提供补贴与支持。
　　5.青年文化精品工程。支持青年文化精品创作推广，支持青年文化创意赛事及文化体验，支持青年文化创意人才培养。每年创作生产一批思想性、艺术性、观赏性俱佳的涵盖各文化类别的青年题材文艺精品。打造一批有影响力的青年网络新媒体产品展播平台，开展全国性青年互联网创新创意活动。在国家级文化、出版类评奖推荐活动中，每年向青年推荐优秀影视、网络、动漫文化作品不少于100小时，图书、报刊文字量不少于200万字，应用类网络游戏不少于3部、网络音乐不少于10首。
　　6.青年网络文明发展工程。深入推进“阳光跟帖”行动，引导广大青年依法上网、文明上网、理性上网，争当“中国好网民”。发展壮大青年网络文明志愿者队伍，持续广泛、强有力、有针对性地发出青年好声音。鼓励支持互联网企业、社会组织、文化机构制作推广符合社会主义核心价值观和青年喜欢的网络新媒体文化产品。加大对中国青年网、未来网、中青在线等青年门户网站、青年公益组织专属网站以及“两微一端”平台的建设扶持力度；加大对青少年新媒体领域社会组织的引导和支持力度，举办网络安全、网络技能、网络文化产品等方面竞赛，发掘、吸引、培养各方面的青年网络人才；加大网络文明队伍指挥协调系统建设力度，开发运行平台，形成管理机制，提升组织效能。倡导网络公益活动，使互联网空间成为青年成长的温馨家园。
　　7.中国青年志愿者行动。全面推行青年志愿者实名注册制度，发挥共青团员示范作用，到2025年实现实名注册的青年志愿者总数突破1亿人。稳步培育青年志愿服务骨干队伍，构建分层分类志愿服务项目库，扩大基层志愿服务组织覆盖，加强激励评价、保险保障等机制建设，形成规模宏大、来源广泛、门类齐全、管理规范的全国青年志愿服务队伍、项目和组织体系，推动青年志愿服务制度化、日常化、便利化开展。坚持以社区为主阵地，广泛开展青年学雷锋志愿服务活动。深入开展大学生志愿服务西部计划，每年选派2万名应届大学毕业生到中西部地区开展志愿服务。坚持立德树人，建立健全学生志愿服务工作体系。深化关爱农民工子女志愿服务专项行动和中国青年志愿者助残“阳光行动”。积极参与并做好重大赛事和会议的志愿服务工作。大力实施中国青年志愿者海外服务计划。
　　8.青年民族团结进步促进工程。实施青年民族团结交流万人计划，每年组织边疆民族地区青年与内地各族青年开展互访、联谊活动，鼓励不同民族青年之间结对子、互帮互助。开展高校“中华文化进校园”活动，每年在200所高校举办图片、影视展和歌舞活动，宣传中华民族形成发展历史，增进中华文化认同，宣传各民族为祖国作出的贡献，增强各族青年学生的中华民族共同体意识。在广大青年中开展民族常识和民族法律法规政策知识大赛。在少数民族流动人口较多的沿海地区和大中城市开展“中华一家亲，可爱城市共同建”活动，为外来少数民族青年融入城市提供帮助。
　　9.港澳台青少年交流工程。进一步扩大内地与港澳青少年之间的交流规模，提升交流质量。继续办好港澳青少年实习实践、体验营、训练营和形式多样的交流考察活动，支持内地与港澳青年组织举办青年论坛，组织青少年开展常态化的结对交流和项目合作，促进相互了解。举办海峡青年论坛、两岸青年社团负责人圆桌会议、两岸青年联欢节等活动。
　　10.青少年事务社会工作专业人才队伍建设工程。到2020年建成20万人、到2025年建成30万人的青少年事务社会工作专业人才队伍，全面参与基层社区社会工作，重点在青少年成长发展、权益维护、犯罪预防等领域发挥作用。在青少年事务社会工作专业教育培训领域，重点扶持发展10家高等教育机构，建立30家具有继续教育资质的培训机构、50家重点实训基地、100家标准化示范单位。制定青少年社会工作服务标准，推动各级团组织以及青少年服务组织和机构设置社会工作岗位，培育青少年事务社会工作服务机构，逐步实现每个“青年之家”综合服务平台至少配备1名青少年事务社会工作专业人才。把青少年社会工作服务纳入政府购买服务指导性目录，组织实施涵盖重点群体、重点领域、重点环节的青少年事务社会工作项目。依法成立青少年事务社会工作领域的社会组织，建设人才队伍管理信息系统平台。建立健全青少年事务领域社区、社会组织、专业社会工作联动机制和社会工作专业人才、志愿者协作机制。完善青少年事务社会工作专业人才培养、评价、使用、激励相关政策配套体系。

　　**四、**组织实施
　　1.加强对规划实施工作的组织领导。在党中央统一领导下，设立推动规划落实的部际联席会议机制，共青团中央具体承担协调、督促职责。各地区各部门要高度重视青年工作，关心、支持青年事业的发展，形成工作合力。县级以上党委和政府建立青年工作联席会议机制，负责推动本规划在本地区的落实，协调解决规划落实中的问题，县级以上团委具体承担协调、督促职责。在规划实施中，要积极回应和解决青年关心的问题，多为青年办实事。
　　2.建立健全青年发展规划体系。各地要以本规划为指导，根据实际编制本地区青年发展规划。注重加强青年发展规划与各地经济社会发展规划及相关专项规划的衔接，更加重视青年发展工作。
　　3.充分发挥共青团维护青年发展权益重要作用。共青团要按照《[中共中央关于加强和改进党的群团工作的意见](https://www.pkulaw.com/chl/71f9cb13088394f4bdfb.html)》和中央党的群团工作会议精神，全面推进自身改革，保持和增强政治性、先进性、群众性，始终紧跟党走在时代前列、走在青年前列，切实代表和维护青年发展权益。同时，要引导青年识大体、顾大局，依法理性表达诉求，自觉维护社会和谐稳定。
　　4.加强服务青年发展阵地建设。大力推进“青年之声”网络互动社交平台建设，依托城乡社区综合服务设施建设“青年之家”综合服务平台，加强网上网下深度融合对接，使其成为服务青年发展的重要阵地。
　　5.保障青年发展经费投入。各级政府将本规划实施所需经费纳入财政预算。动员社会力量，多渠道筹集资金，支持青年发展。
　　6.营造规划实施良好社会环境。大力宣传党和国家关于青年工作的重大战略思想和方针政策，宣传关心青年就是关心未来的理念，宣传青年先进典型和成功经验，形成全社会关心、支持青年发展的良好社会氛围，形成推动本规划实施的强大合力。
　　7.建立规划实施情况监测评估机制。对本规划实施情况进行年度监测、中期评估和终期评估，制定和调整促进青年发展政策措施，推动本规划实现。规范和完善与青年发展有关的统计指标体系，收集、整理、分析相关数据和信息，建立和完善中央、省（自治区、直辖市）两级青年发展监测数据库。

© 北大法宝：（[www.pkulaw.com](https://www.pkulaw.com/)) 专业提供法律信息、法学知识和法律软件领域各类解决方案。北大法宝为您提供丰富的参考资料，正式引用法规条文时请与标准文本核对。
欢迎查看所有[产品和服务](https://www.pkulaw.com/helps/2.html)。法宝快讯：[如何快速找到您需要的检索结果？](https://www.pkulaw.com/helps/70.html)[法宝](https://www.pkulaw.com/helps/69.html)V6有何新特色？

扫描二维码阅读原文

**原文链接：**<https://www.pkulaw.com/chl/c9ba542878e8ad32bdfb.html>
